# Targeting the value of targeted therapy

**DOI:** 10.18632/oncotarget.21596

**Published:** 2017-10-07

**Authors:** Joseph C. Del Paggio, Richard Sullivan, Christopher M. Booth

**Affiliations:** Christopher M. Booth: Departments of Oncology and Public Health Sciences, Queen’s University, Kingston, ON, Canada

**Keywords:** systemic therapy, cancer, value, health policy, clinical trials

Within the last two years, the Federal Drug Administration (FDA) in the United States has approved 17 drugs for a spectrum of advanced hematological and solid cancers; of these drugs, approximately 95% (i.e., all but 1 drug) fall within the category of targeted anticancer agents [1]. Within the spectrum of targeted anti-cancer therapy lies a paradox: costs of therapies are skyrocketing, yet the magnitude of benefit is decreasing [2]. Moreover, the novelty of these agents, as measured by the costs incurred through research and development, lies in stark disconnect from the market values of these drugs [3]. In this context, the concept of value (i.e., the relationship between cost and outcome) has become the crux of the conversation about new cancer therapies. As the annual global market for cancer drugs is expected to reach $150 billion dollars in the very near future [3], this conversation is a necessity for health systems globally.

Within the value equation it is relatively simple to derive costs/price; what is more challenging is the act of quantifying benefit. Several frameworks have been recently developed to address this very challenge. Both the American Society of Clinical Oncology Value Framework (ASCO-VF) [4] and the European Society for Medical Oncology Meaningful Clinical Benefit Scale (ESMO-MCBS) [5], among others, seek to objectify an oncological ‘bone of contention’: the extent to which a new drug offers “meaningful clinical benefit”. Although quite different in their derivation, both frameworks attempt to transform the combination of survival gains, drug toxicities, and quality of life improvements into a simple score. But, in attempting to do so, it has become obvious that developing a unified concept of benefit poses a challenge.

Three recent cohort studies have shed light on the congruity between the ASCO-VF and the ESMO-MCBS. The first, by Becker et al., assessed 55 trials (10 phase II and 45 phase III) that formed the basis of 55 FDA drug approvals, 75% of which took place after 2010 [6]. Cheng et al. expanded their analysis to 109 trials from 2006 to 2015 consisting of drugs approved not only by the FDA, but also by the European Medicines Agency and Health Canada [7]. Finally, our group assessed a cohort of 109 RCTs with statistically significant results in favour of the experimental arm published during 2011-2015, of which only a minority (10%) formed the basis for FDA drug approval [8]. Importantly, 80% of the experimental arms contained targeted anticancer therapies, in keeping with the contemporary nature of the cohort. Despite the obvious differences in trial populations, the results of each of the three cohort studies are remarkably consistent: there is only weak-to-moderate correlation between these two major value frameworks. The implication is that, despite similarities between their components, these tools offer different perspectives on what drugs confer “benefit”.

How do these frameworks’ constructs of benefit fit in with the other half of the value equation—cost? Becker et al. and our own group have used the framework outputs to correlate the ASCO-VF and the ESMO-MCBS with the average wholesale price of the experimental arms [6, 8]. In both studies, there was no observed correlation between the magnitude of benefit conferred by a new drug and the cost. This lack of correlation between outcomes and cost follows much of what has been hitherto identified on the subject, only now using expert-opinion and evidenced-based framework outputs of benefit. It is notable that within our own cohort, we identified a significant *negative* correlation between framework scores and incremental costs (i.e., monthly cost of the experimental arm - monthly cost of the control arm) for both the ASCO-VF and the ESMO-MCBS [8].

The disconnect observed within and between these value frameworks is analogous to the very nature of targeted molecular therapy: given cancer’s highly molecular heterogeneity, it should not be of surprise that most targeted therapies benefit only a select group of patients. These value frameworks will, therefore, naturally fall short in appreciating the ‘signals’ of benefit in particular subsets of the trial population, since such an appreciation was neither their design nor their intent. Despite their limitations, the existing frameworks have initiated an important conversation within the communities of clinical care, research, policy, and drug development. We must prioritize our efforts on delivery of high-value therapies that offer real benefit to our patients in the context of sustainable health systems.
